# Numerical Inconsistencies in “Physician-Staffed Ambulance Deployment: Comparative Response Time Analysis from a Slovak Pilot Project”

**DOI:** 10.5811/westjem.67945

**Published:** 2026-10-18

**Authors:** Adrián Fabiny, Dušan Sysel, Teodor Bachleda

**Affiliations:** * Comenius University in Bratislava, Faculty of Medicine, Clinic of Emergency Medicine and Disaster Medicine, Bratislava, Slovak Republic; † Comenius University in Bratislava, Jessenius Faculty of Medicine in Martin, Department of Nursing, Bratislava, Slovak Republic

Dear Editor:

We read with interest the study by Sedlak et al on a physician-staffed rendezvous (RV) model in Košice.^1^ The question is timely and the dataset substantial, and we welcome this contribution. In a constructive spirit, we raise several numerical inconsistencies that warrant correction, and one interpretive point. These inconsistencies appear to be transcription or typesetting errors that do not, in our reading, alter the direction or significance of the principal findings; we note them to support the accuracy of the record.

First, in Table 1, the standard deviation for RLP Západ response time postintervention is 34.63 minutes, while the reported minimum and maximum are 2 minutes and 51 minutes. For any sample bounded within this range, the maximum attainable standard deviation is approximately (51–2)/2 ≈ 24.5 minutes. The reported value cannot derive from these data and is likely a transcription error.

Second, two maxima in Table 1 appear implausible: The on-scene interval for RV Sever lists a maximum of 12600 minutes (other maxima in this block range ~126–160), and the crew-availability interval lists 19000 mins. A genuine value of 19000 in a sample of 488 would raise the mean by tens of minutes; since the reported mean (49.58) is consistent with a maximum near 190, these appear to be misplaced decimal points.

Third, in Table 4 the 95% CI for the postintervention indicator is printed as [−1.1013, −0.525]. Recalculation from the reported coefficient and standard error (−0.7692 ± 1.96 × 0.124) yields [−1.012, −0.526], matching the value cited in the abstract and discussion sections (−1.013 to −0.525). The tabulated lower bound appears to contain an extra digit.

Fourth, also in Table 4, the intercept coefficient (9.3295) and standard error (0.199) yield *t* ≈ 46.9 rather than the printed 49.6. The remaining rows are internally consistent, suggesting an isolated error in this row. Furthermore, for dispatch priority “M,” the reported interval [0.075, 0.386] excludes the null value, which is incompatible with the accompanying *P* = .19; the correct interval (0.1552 ± 1.96 × 0.118) is approximately [−0.076, 0.386], the lower bound apparently carrying an incorrect sign. This does not affect interpretation, as the authors treat this covariate as nonsignificant.

Fifth, the results section states that the largest response-time improvements were “in Košice 2 and Košice 3 (absolute reductions of 2.64 and 2.15 minutes, and effect sizes r_e_ = 0.616 and 0.419, respectively).” Table 3, however, attributes the 2.15-minute reduction and r_e_ = 0.419 to Košice 2, and the 2.64-minute reduction and r_e_ = 0.616 to Košice 3. The values appear transposed. We also note that the Table 3 footnote defines r_e_ as “random effects,” whereas the methods section defines it as the rank-biserial correlation; and the regression reports *N* = 10,687 against an analytic cohort of 11,094, a difference of 407 cases that is not explained in the text.

Beyond these corrigible items, we offer one interpretive observation. The improvement in time-to-crew-availability is concentrated in the RV unit (Sever: 60.78 → 49.58 minutes), whereas the two retained physician-staffed transport units show the opposite trend over the same period (Staré Mesto 57.36 → 64.25; Západ 57.91 → 62.67). This redistribution is reported in the results section but is not reflected in the abstract or conclusion, which characterize the availability gain in system-level terms. Because the study does not directly compute a net system-wide availability balance, we suggest the conclusion on availability be framed with this trade-off made explicit. We emphasize that the response-time findings are unaffected: as per Table 1, response time improved across all three units, and the authors’ conclusion on that outcome is well-supported.

We thank the authors for an informative analysis and hope these observations are received in the collegial spirit intended.
